# Study on Anti-Inflammatory Effect and Major Anti-Inflammatory Components of PSORI-CM02 by Zebrafish Model

**DOI:** 10.1155/2020/5604654

**Published:** 2020-05-28

**Authors:** Xiaohong Yuan, Leyu Huang, Jiaqi Lei, Yingxin Long, Cong Li

**Affiliations:** ^1^The Second Affiliated Hospital of Guangzhou University of Chinese Medicine, Guangzhou 510120, China; ^2^Guangdong Provincial Academy of Chinese Medical Sciences, Guangzhou 510006, China

## Abstract

PSORI-CM02 is an optimization formula of PSORI-CM01, which is a clinical herbal formula for the treatment for psoriasis in the Guangdong Provincial Hospital of Chinese Medicine. Previous research indicates that it plays a critical role in anti-inflammation and immunoregulation. Rhizoma smilacis glabrae (RSG) is one herbal medicine of PSORI-CM02, whose effective anti-inflammatory component is astilbin. This study aims to test the anti-inflammatory and immunoregulation effects of astilbin as well as RSG in PSORI-CM02, and we, respectively, used the CuSO_4_-induced neutrophil-specific transgenic zebrafish model *Tg(mpx: EGFP)* and the melanin allele mutated Albino strain zebrafish model to visualize the effects of neutrophil recruitment and macrophage phagocytosis. Our data indicated that both PSORI-CM02 and astilbin had anti-inflammatory effects, leading to a reduction in the recruitment of neutrophils and promotion in macrophage phagocytosis. Nevertheless, the negative liquor of Rhizoma smilacis glabrae (PSORI-CM02 without RSG) also had anti-inflammatory and promoting macrophage phagocytosis effects. The results revealed the formula excluding RSG also had anti-inflammatory and immunoregulation effects, which demonstrated that RSG was not the major anti-inflammatory herbal medicine in PSORI-CM02. Similarly, astilbin was not the major anti-inflammatory active ingredient in the formula. The anti-inflammatory and the promotion of macrophage phagocytosis effect of PSORI-CM02 *in vivo* zebrafish were the results of multiple component interaction, which was the common characteristic of the Chinese medicine compound.

## 1. Introduction

Inflammation is a complicated biological and pathophysiological cascade of responses to infections and injuries. Inflammatory mechanisms are also closely linked with many diseases including cardiovascular disease, rheumatoid arthritis, inflammatory bowel disease, Alzheimer's disease, and even cancer [1, 2]. The inflammatory process is mediated by diverse inflammatory cells, including neutrophils, macrophages, eosinophils, and mononuclear phagocytes [3]. Under inflammatory conditions, neutrophils in the blood migrate towards the harmed sites [4]. Also, macrophages are major immune cells in the innate immune system and play essential roles in inflammation and directly counteract harmful external stimuli. Activated macrophages play important roles in host defenses against infection with pathogens, to initiate phagocytic activities and promote inflammatory responses via producing various inflammatory mediators [5–7]. Phagocytosis of pathogens, apoptotic cells, and debris is a critical feature of macrophage function in host defense and tissue homeostasis [8]. Therefore, monitoring the number of neutrophils in animals and examining macrophage phagocytosis are effective methods for evaluating inflammatory responses. The occurrence and development of many diseases are related to immunity and inflammation. Consequently, drug research with immune and anti-inflammatory effects has always been a hot topic in the field of research.

Zebrafish is highly similar to mammals in morphology and physiology, and its embryo's optical transparency can make noninvasive and dynamic imaging of the inflammation *in vivo* [9]. Furthermore, the generation of transgenic zebrafish expressing the neutrophil-specific green fluorescent protein (GFP) has made it possible to observe neutrophil behavior *in vivo* [4]. The removal of neutrophils by reverse migration as well as by apoptosis and macrophage uptake has also been observed in the process of inflammation resolution [10, 11]. Macrophages are not only participating in the removal of cellular debris which is produced during tissue remodeling, but also promptly and effectively clear cells that have undergone apoptosis. The major role of macrophages is to clear the interstitial environment of extraneous cellular material [12]. Consequently, in the second experiment, the melanin allele mutated Albino strain zebrafish were injected with ink intravenously to observe macrophage phagocytosis, so that the effect of the drugs on macrophage phagocytosis could be evaluated. Finally, the anti-inflammatory effects of the drugs were evaluated by these two zebrafish models.

Chinese medicine compound is the primary form for the clinical application of Chinese medicine, usually composed of multiple kinds of Chinese herbal medicines, which plays a pharmacodynamic role in the human body through mutual coordination or restriction [13]. PSORI-CM02 is an optimization formula of PSORI-CM01, a classic prescription of a national famous doctor Guo-Wei Xuan, which has been used for psoriasis vulgaris for more than twenty years in the Guangdong Provincial hospital [14]. PSORI-CM02 consists of Rhizoma curcumae, Radix paeoniae rubra, *Sarcandra glabra*, Rhizoma smilacis glabrae, and *Fructus mume*, which is proved to have anti-inflammatory effects and improve immunity [15]. RSG, which is a traditional Chinese medicine in PSORI-CM02, has been reported to have an anti-inflammatory effect, and its major effective active component is astilbin [16]. PSORI-CM02 also possesses anti-inflammatory activities; however, the major anti-inflammatory components are still unclear. Here, we sought to clarify whether RSG is the main traditional Chinese medicine that has an anti-inflammatory role and astilbin is the main active component of anti-inflammation in PSORI-CM02. To this end, two pathological models of zebrafish were established: the copper sulfate-induced zebrafish inflammation model and the zebrafish macrophage phagocytosis function evaluation model. Furthermore, related pharmacodynamic experiments were conducted to evaluate the anti-inflammatory effect and correlation among astilbin, RSG, and PSORI-CM02.

## 2. Materials and Methods

### 2.1. Materials

#### 2.1.1. Instruments and Reagents

Six-well plate (Nest Biotech, China); dissecting microscope (SZX7, OLYMPUS, Japan); camera attached to a dissecting microscope (VertA1, China); precision electronic balance (CP214, OHAUS, America); electric focus continuous zoom fluorescence microscope (AZ100, Nikon, Japan); methylcellulose (Sigma, China); dimethyl sulfoxide (DMSO) (Sigma, China); neutral red (Sigma, China) were used.

#### 2.1.2. Experimental Animals

In this experiment, two zebrafish strains, transgenic neutrophil fluorescent zebrafish *Tg(mpx: EGFP)* and melanin allele mutant albino zebrafish, were used for natural mating and reproduction. Each experimental group consisted of 30 zebrafish, which were purchased from Hunter Biotechnology, Inc. Also, three-day postfertilization (dpf) zebrafish were used in this study. All zebrafish were maintained at 28°C in fish water (200 mg of Instant Ocean Salt in each liter of reverse osmosis water, pH 6.9–7.2, conductivity 480–510 *μ*S/cm, and hardness: 53.7–71.6 mg/L CaCO_3_) under 14 h/10 h light/dark cycle). The license number of laboratory animals is SYXK (Zhejiang) 2012–0171. All experiments were performed in accordance with the requirements of the international AAALAC certification.

### 2.2. Preparation of Experimental Drugs

#### 2.2.1. Extraction of PSORI-CM02 Liquor

According to the prescription, the herbs were extracted three times after soaking with 8 times water for 30 minutes, 1 hour for the first time and 45 minutes for the second and third time; it was then filtered by a gauze, combined with three times of extracting liquor and finally concentrated into the extract. The 20 mg/mL mother liquor was prepared with 10% DMSO aqueous solution before using. This is drug A which contained astilbin: 3.9216 mg/g.

#### 2.2.2. RSG Negative Liquor (PSORI-CM02 Negative Liquor)

The same operation as mentioned in Section 2.2.1 was carried out, but without RSG. 20 mg/mL mother liquor was prepared with 10% DMSO aqueous solution before using. This is drug B.

#### 2.2.3. Astilbin Solution

Astilbin was weighed and used to prepare 20 mg/mL mother liquor with DMSO before using it. This is drug C.

### 2.3. Establishment of Zebrafish Inflammation Model

The 3-day postfertilization zebrafish (3 dpf) were incubated in six-well plates and treated with 10 *μ*M CuSO_4_ for 2 hours at 28°C, and image analysis was performed by using advanced image processing software to calculate the number of neutrophils (*n*) at the inflammatory site of the zebrafish. The number was compared between the model control group (28) and the normal control group (6), indicating that the copper sulfate-induced zebrafish inflammation model was established successfully (*p<*0.05).

### 2.4. Determination of the Maximum Tolerated Concentration of Drugs A, B, and C in the Zebrafish Inflammation Model

Before the formal experiment, the toxicity and maximum tolerated concentration of the experimental drugs should be investigated. Toxicity of drugs A, B, and C based on morphological changes and death numbers of neutrophil-specific transgenic zebrafish *Tg(mpx: EGFP)* was evaluated.

At three-day postfertilization (dpf), 510 transgenic zebrafish were randomly selected and divided into 17 groups and placed in six-well plates. Each well (experimental group) was treated with 30 zebrafish and incubated in an incubator at 28°C. The test was started by exposing the zebrafish to different concentrations of drugs A, B, and C dissolved in water, respectively, with a capacity of 3 mL per well. They were divided into the blank control group, the model control group treated with 10 *μ*M CuSO_4_ for 2 hours, and drugs A, B, and C groups. Each experimental drug was given at 5 concentrations (drug A: 250, 500, 1000, 1500, and 2000 *μ*g/mL; drug B: 250, 500, 1000, 1500, and 2000 *μ*g/mL; drug C: 125, 250, 500, 1000, and 2000 *μ*g/mL). After one hour of treatment, except for the blank control group, the other groups were given 10 *μ*M CuSO_4_ to establish the inflammation model of transgenic zebrafish. Two hours later, morphological changes and mortality of zebrafish were observed to determine the maximum tolerance concentration of the three drugs to neutrophil-specific transgenic zebrafish.

### 2.5. Evaluation of the Anti-Inflammatory Effects of Drugs A, B, and C Using the Zebrafish Inflammation Model

A total of 360 zebrafish were randomly assigned into twelve groups with 30 zebrafish in each group: a blank control group, a model control group exposed to 10 *μ*M CuSO_4_ for 2 h, a positive control group (80 *μ*M indometacin), and other drug experimental groups. All zebrafish were incubated in six-well plates in an 28°C incubator, with a capacity of 3 mL per well. Subsequently, the operations were performed according to those mentioned in Section 2.4. The dose concentrations of drug A were 222, 667, and 2000 *μ*g/mL and those of drugs B and C were 111, 333, and 1000 *μ*g/mL. After two hours of administration, 10 zebrafish were randomly chosen from each group and photographed under the continuous zoom fluorescent microscope with electric focusing. The anti-inflammatory activities of drugs A, B, and C on CuSO_4_-induced inflammatory zebrafish were evaluated by statistical analysis of the number of neutrophils. The ratio of inflammatory regression effect was calculated based on the number of neutrophils. The calculation formula is given as follows:

(1) $$\begin{array}{c} \text{Inflammatory regression }\left(\%\right)=\frac{N\left(\text{model control group}\right)-N\left(\text{test group}\right)\;}{N\left(\text{model control group}\right)}\times 100\%. \end{array}$$

### 2.6. Establishment of Zebrafish Macrophage Function Evaluation Model

The experiment was carried out using a zebrafish macrophage phagocytosis evaluation model. Ink was administered intravenously to 3 dpf melanin allele mutant Albino strain zebrafish incubated in a 28°C incubator for 48 hours, and then the zebrafish were observed and photographed by an anatomical microscope. The number of macrophages which swallowed ink was analyzed and counted by advanced image processing software to evaluate the effect of the samples.

### 2.7. Determination of the Maximum Tolerated Concentration (MTC) of Drugs A, B, and C in the Zebrafish Macrophage Phagocytosis Model

At three-day postfertilization (3 dpf), 510 melanin allele mutated Albino strain zebrafish were randomly divided into 17 groups in the same way as given in Section 2.4. They were placed in 6-well plates with a capacity of 3 mL per well. Each well (each concentration group) consisted of 30 zebrafish that were injected with ink intravenously to establish the macrophage phagocytosis model. The zebrafish were given different concentrations of water-soluble drugs A, B, and C, respectively (drug A concentration: 250, 500, 1000, 1500, and 2000 *μ*g/mL; drug B concentration: 250, 500, 1000, 1500, and 2000 *μ*g/mL; drug C concentration: 62.5, 125, 250, 500, and 1000 *μ*g/mL). Simultaneously, the normal control group and the model control group were also set up. The normal control group was treated with system water, while the model group was only injected with ink intravenously without giving the drug. Morphological changes and mortality of zebrafish were observed by using an anatomical microscope after incubation in an incubator at 28°C for 48 hours. Subsequently, the toxicity of these three drugs in the phagocytic function model was analyzed to determine the MTC of drugs A, B, and C to the melanin allele mutated zebrafish.

### 2.8. Effects of Drugs A, B, and C on the Phagocytic Function of Zebrafish Macrophages

At 3 dpf, 360 melanin allele mutation zebrafish were randomly selected and transferred into six-well plates. To establish the macrophage phagocytosis function model, 30 zebrafish in each well (each concentration group) were injected with ink intravenously. According to the results of the MTC experiment, the concentrations of drug A were 56, 167, and 500 *μ*g/mL, the concentrations of drug B were 56, 167, and 500 *μ*g/mL, the concentrations of drug C were 111, 333, and 1000 *μ*g/mL, and the concentration of the positive control drug Bailing capsule was 15.6 *μ*g/mL. At the same time, the normal control group and the model control group were set up. The capacity of each well is 3 mL. After 24 hours of incubation in an incubator at 28°C (i.e., 3 dpf to 4 dpf), zebrafish were dyed *in vivo* with a neutral red solution. After dyeing, 10 zebrafish were randomly selected from each group to observe, photograph, and preserve pictures under a microscope. Nikon NIS-Elements D mean 3.10 advanced image processing software was used to statistically analyze the zebrafish with swallowed ink. The number of macrophages (N) in zebrafish was expressed by mean ± SE, and the effect of samples on the phagocytic function of macrophages in zebrafish was evaluated by statistical significance. The formula for calculating the effect of the tested samples on the phagocytosis of zebrafish macrophages is as follows:

(2) $$\begin{array}{c} \text{Enhancement of macrophage phagocytosis}\left(\text{\%}\right)=\frac{N\left(\text{test group}\right)-N\left(\text{model control group}\right)}{N\left(\text{model control group}\right)}\times 100\text{\%}. \end{array}$$

### 2.9. Statistical Analysis

All data are presented as mean ± standard error (SE). Significant differences among groups were determined using Dunnett's *t*-test and variance analysis. A value of *p<*0.05 was accepted as an indication of statistical significance.

## 3. Results

### 3.1. MTC of Drugs A, B, and C in the Zebrafish Inflammation Model

Prior to testing for the anti-inflammatory activity of drugs A, B, C, the toxicological evaluation of the experimental drug was carried out. Two hours later, the number of death and toxicity of zebrafish were observed and recorded to evaluate the toxicity of drugs A, B and C and determine the maximum dosage concentration (MTC). The results showed that zebrafish were not dead in the following drug concentration ranges (A: 250–2000 *μ*g/mL; B: 250–2000  *μ*g/mL; C: 125–2000 *μ*g/mL) (Table 1). However, at doses of 1500 and 2000 *μ*g/mL of drug B, zebrafish are insensitive to stimulation, and their toxicity reaction is more serious than that of the model control group. Accordingly, the maximum dose concentration of drug B is 1000 *μ*g/mL. When the concentrations of drug C were 1500 and 2000 *μ*g/mL, drug precipitation occurred, so the maximum dose concentration of drug C was set at 1000 *μ*g/mL. According to the experiment results, the following concentrations of drugs, drug A: 222, 667, and 1000*μ*g/mL; drug B: 111, 333, and 1000 *μ*g/mL; drug C: 111, 333, and 1000 *μ*g/mL, were selected for subsequent experiments.

### 3.2. Evaluating the Anti-Inflammatory Effects of Drugs A, B, and C by the Zebrafish Inflammation Model

**Figure 1:**
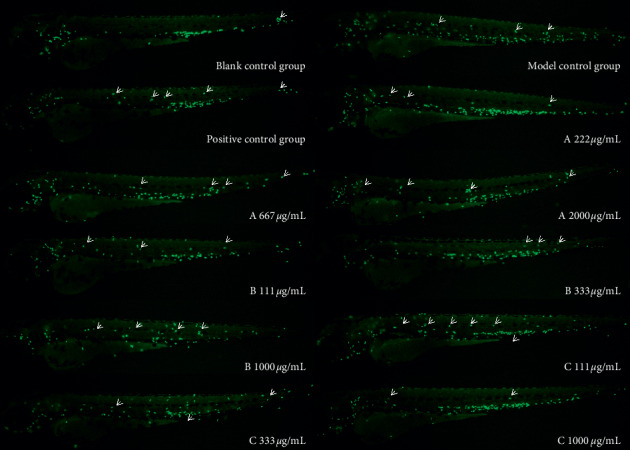
Phenotype map of the effects of each experimental group on zebrafish inflammation. Fluorescence microscopy was used to reveal neutrophils in *Tg(mpx: EGFP)* larvae. The number of neutrophils in the arrow pointing area was counted.

**Figure 2:**
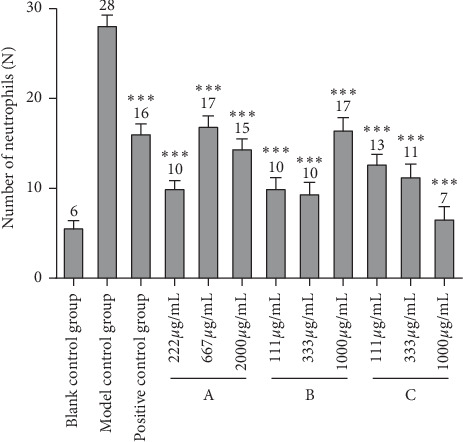
Effects of experimental groups on zebrafish inflammation (number of neutrophils). Neutrophils were detected at the inflammation site after CuSO_4_ treatment for 2 h. The number of neutrophils was counted at the inflammation site. *N* = 10, comparing to the model control group: ^*∗∗∗*^*p<*0.001.

**Figure 3:**
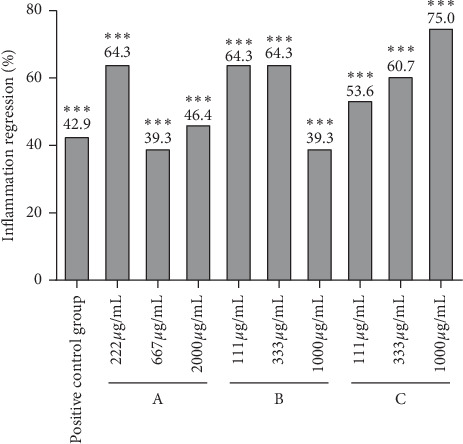
Regressive effect of inflammation on each experimental group. All zebrafish larvae were incubated in six-well plates, and the capacity of each well was 3 mL. Except for the blank control group, the aqueous solution was given to drugs A, B, and C and indometacin for one hour, respectively. Neutrophils were detected at 2 h after treatment with 10 *μ*M CuSO_4._ The positive control was treated with 80 *μ*M indometacin. A: PSORI-CM02 was used at concentrations of 222, 667, and 2000 *μ*g/mL. B: PSORI-CM02 negative drug was used at concentrations 111, 333, and 1000 *μ*g/mL. C: astilbin was used at concentrations 111, 333, and 1000 *μ*g/mL. The number of neutrophils was counted and statistically analyzed. The ratio of inflammatory regression effect was calculated based on the number of neutrophils. *N* = 10, ^*∗∗∗*^*p<*0.001.

As copper sulfate can induce auditory cell injury and lead to an inflammatory reaction, neutrophils accumulate at the site of inflammation, thereby establishing an inflammatory cell model. In this experiment, a CuSO4-induced zebrafish inflammation model was used to evaluate the anti-inflammatory effects of the three drugs. As shown in Figure 1, many fluorescent neutrophils were recruited to the site of inflammation of the CuSO4-induced zebrafish. The average number of fluorescent neutrophils in the inflammatory site of CuSO4-induced zebrafish model was 28 ± 1, while that of CuSO4-induced juvenile fish treated with 222, 667, and 2000 *μ*g/mL of drug A was 10 ± 1, 17 ± 1, and 15 ± 1, respectively (Table 2). Similarly, the mean of fluorescent neutrophils in inflammatory sites of zebrafish treated with 111, 333, and 1000 *μ*g/mL drug B was 10 ± 1, 10 ± 1, and 17 ± 1, respectively, and the number of treated with 111, 333, and 1000 *μ*g/mL drug C was 13 ± 1, 11 ± 1, and 7 ± 1, respectively. The rate of inflammation regression is given in Table 2. The results of the experiment (Figure 2 and Figure 3) indicated that the three drugs A, B, and C all had different degrees of anti-inflammatory activity, and the anti-inflammatory effect of drugs A and B at low concentration was better than that at high concentration and were better than that of the positive control drug. The anti-inflammatory effect of drug C was also better than that of the positive control drug in a dose-dependent pattern.

### 3.3. MTC of Drugs A, B, and C in the Zebrafish Macrophage Phagocytosis Model

As given in Table 3, zebrafish treated with 250 and 500 *μ*g/mL of drugs A and B had no obvious toxic phenotype. The mortality of drug A at concentrations of 1000, 1500, and 2000 *μ*g/mL was 23.3%, 46.7%, and 96.7%, respectively. At 250 and 500 *μ*g/mL of drug B, the zebrafish did not die. Besides, the mortality rates were 70%, 100%, and 100% at 1000, 1500, and 2000 *μ*g/mL, respectively, so the concentration of MTC of drug A and drug B was 500 *μ*g/mL. Moreover, the dose of 62.5–1000 *μ*g/mL of drug C did not cause any toxic effects in the developmental stage of zebrafish, but when the dissolution concentration of drug C is more than 1000 ug/mL, it precipitates, so the maximum dose of drug C was 1000 *μ*g/mL.

Based on the above experimental results, the following concentrations: drug A: 56, 167, and 500 *μ*g/mL; drug B: 56, 167, and 500 *μ*g/mL; drug C: 111, 333, and 1000 *μ*g/mL were selected for subsequent experiments.

### 3.4. The Promotion of Phagocytosis of Zebrafish Macrophages by Drugs A, B, and C

**Figure 4:**
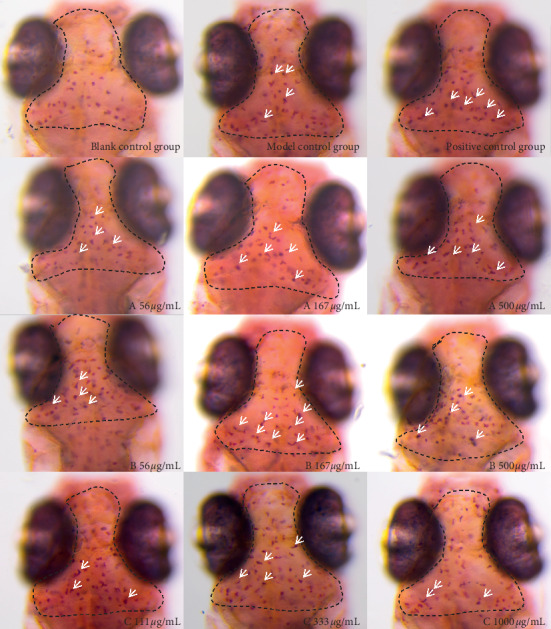
Phenotype of ink phagocytosis by macrophages in the zebrafish brain. Statistical area is in the black dotted line frame. The white arrow indicates the macrophage that has swallowed ink. Because it has swallowed ink, the color of macrophage is darker than that of normal macrophage.

**Figure 5:**
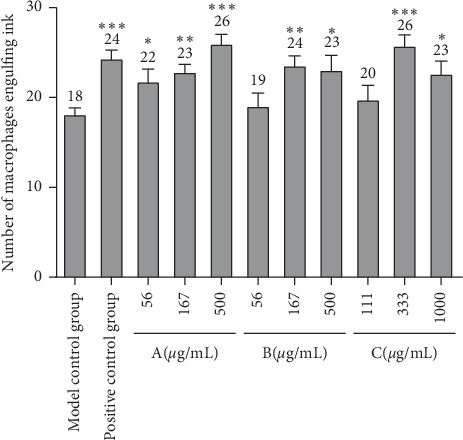
Number of macrophages swallowing ink in the zebrafish brain in each experimental group. All zebrafish larvae except the blank control group were injected with ink. The positive control was injected with the Bailing capsule solution (15.6 *μ*g/mL). A: drug A was used at concentrations of 56, 167, and 500 *μ*g/mL. B: drug B was used at concentrations of 56, 167, and 500 *μ*g/mL. C: drug C was used at concentrations of 111, 333, and 1000 *μ*g/mL. The number of macrophages swallowing ink was counted and statistically analyzed. The ratio of promoting macrophage phagocytosis effect was calculated based on the number of macrophages swallowing ink. *N* = 10, ^*∗*^*p<*0.05, ^*∗∗*^*p<*0.01, and ^*∗∗∗*^*p<*0.001.

**Figure 6:**
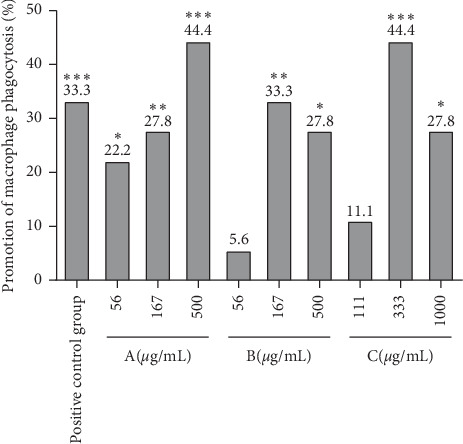
Promoting macrophages phagocytosis effect of inflammation in each experimental group. All zebrafish larvae except the blank control group were microinjected with ink. The positive control was injected with an equal volume of the Bailing capsule solution. A: drug A was used at concentrations of 56, 167, and 500 *μ*g/mL. B: drug B was used at concentrations of 56, 167, and 500 *μ*g/mL. C: drug C was used at concentrations of 111, 333, and 1000 *μ*g/mL. The phagocytosis of macrophages was observed after 24 hours of incubation, and the number of macrophages (N) which swallowed ink in zebrafish was counted. The ratio of promoting macrophage phagocytosis effect was calculated based on the number of macrophages swallowing ink. *N* = 10, ^*∗*^*p<*0.05, ^*∗∗*^*p<*0.01, and ^*∗∗∗*^*p<*0.001.

After establishing the phagocytic function model of zebrafish macrophages and giving the drugs for 24 hours, the number of macrophages phagocytizing ink was observed from the maxillary to the two ears of zebrafish, as shown in Figure 4. The number of macrophages phagocytizing ink in the positive group and low, medium, and high dose group of drugs A, B, and C is shown in Figure 5, and the macrophages promoting phagocytosis is shown in Figure 6 and Table 4. The results indicated that the three drugs A, B, and C all promoted the phagocytosis of macrophages at the experimental concentration. The phagocytosis promoting effect of drug A on zebrafish macrophages was positively correlated with the dose. The effect of drug B and drug C was the best in the medium dose, while the effect of drug A in the high concentration group and drug C in the medium concentration group was better than that of the positive control group. The effect of drug B in the medium concentration group was similar to that in the positive control group.

## 4. Discussion

“Multicomponent, multichannel, and multitarget” are the characteristics and advantages of Chinese medicine compound treatment of illness [17]. The efficacy of the TCM compound is usually the result of the synergistic effect of multiple components, which is also reflected in the results of this experiment. PSORI-CM02 is a representative herbal formula for psoriasis in our hospital. Many clinical and experimental studies have been carried out in the early stage, and the results of anti-inflammatory research indicated that PSORI-CM02 could inhibit the mRNA expression of proinflammatory cytokines such as TNF*α*, IL-6, and IL-17 and reduce protein levels in serum [18]. RSG is a traditional Chinese medicine in PSORI-CM02, which has been reported to have anti-inflammatory activities, and its main biological active ingredient is astilbin [16, 19]. To explore the correlation between RSG and PSORI-CM02 in the anti-inflammatory effect, we conducted this experiment.

Inflammation is a physiological reaction of the innate immune system which aims to reduce the pathogenic factor that leads to tissue injury and/or minimize external influence, to induce appropriate wound healing, and to restore tissue homeostasis [20]. As the inflammatory response is initiated, neutrophils and lymphocytes are recruited at an early phase, followed by macrophage activated intensely at the later stages [1]. However, if the body is unable to resolve the acute inflammatory response, it would develop into chronic and persistent inflammation [21]. It has been reported that neutrophils were involved in the adaptive immune response to resolve the chronic inflammation and also implicated the involvement of monocytes/macrophages in acute inflammatory response [22]. For the purpose of tackling the inflammation, neutrophils must be cleared effectively either by undergoing apoptosis and macrophage engulfment or by leaving the inflammation area through reverse migration [23–25].

In the neutrophil experiment, the neutrophils were damaged by copper sulfate to fluoresce the auditory cells of zebrafish, which caused the inflammatory reaction. The number of neutrophils in the inflammatory site (the lateral position of zebrafish, i.e., near the auditory cells of zebrafish) was calculated. In the macrophage phagocytosis of ink experiment, the macrophages were stained with neutral red and the number of macrophages in the head of zebrafish was counted. These two experiments were all operated under the microscope. The operation is simple, and the experimental results are intuitionistic. The number of neutrophils and macrophages engulfing ink in the inflammatory site can be obtained accurately. Consequently, investigating the number of neutrophils in the inflammation site and macrophage phagocytosis could act as effective methods for evaluating the anti-inflammatory activity of drugs.

In the recent years, as a model organism, zebrafish have many advantages, such as transparent embryo, small size, convenient feeding, rapid development, simultaneous and direct observation of the effects of drugs on multiple tissues and organs, and small sample dosage. They are widely used in toxicity and pharmacodynamics research [26]. By using the transparent characteristics of the zebrafish embryo, the behavior of inflammatory cells can be observed through the microscope and the anti-inflammatory effect of drugs can be investigated. In the present experiment, CuSO_4_ was used to induce auditory cell injury in zebrafish to establish an inflammation model. The experimental results showed that drugs A, B, and C could inhibit the aggregation of neutrophils to inflammatory sites. The anti-inflammatory effect of drugs A and B was better in the low dosage, and the anti-inflammatory effect of both drugs was equal, while the anti-inflammatory effect of drugs C was better in the high dosage. The evaluation of the promoting effects of drugs A, B, and C on the phagocytosis of macrophages showed that the drugs A, B, and C all had promoting effects on the phagocytosis of macrophages.

Our results indicate that PSORI-CM02 had anti-inflammatory activity even without RSG, and its anti-inflammatory activity was comparable. The anti-inflammatory activity of astilbin (drug C) was positively correlated with the dosage, while inflammation subsidence of 111 ug/mL was only 53.6%, which was lower than 64.3% of low-concentration A drug (0.8706 ug/mL containing astilbin). Even without RSG, PSORI-CM02 still had the same anti-inflammatory effect, which showed that astilbin had an anti-inflammatory effect, but it was not the main effective ingredient of the anti-inflammatory effect in PSORI-CM02. In the evaluation experiment of macrophage phagocytosis function, the promoting effect of drug A was stronger than that of drug B, but the difference was not obvious. However, 500 ug/mL drug A (astilbin content is 1.9608 ug/mL) had the same promoting effect as 333 ug/mL astilbin. Similarly, the experiment also provided evidence that astilbin was not the main active ingredient in PSORI-CM02, but only one of the effective ingredients, reflecting the characteristics synergistic effect of multicomponent of the Chinese medicine compound.

In the pre-experiment, an assay of maximum administration dosage of drugs A, B, and C on zebrafish was conducted. In the anti-inflammatory model, drugs A, B, and C all have small side effects. The maximum administration dosage of drug A was 2000 *μ*g/mL and that of drugs B and C was 1000 *μ*g/mL. Zebrafish had no adverse reactions with the three drugs at MTC concentrations. In the macrophage phagocytosis model, 500 *μ*g/mL of drugs A and B and 1000 *μ*g/mL of C were chosen as the maximum administration dosage (consider solubility factor). In the formal experiment, multiple concentration groups were established within the range of MTC concentrations to evaluate the drug safety of the anti-inflammatory model and the macrophage phagocytosis model, respectively.

In conclusion, the present study is the first to study the anti-inflammatory effect of PSORI-CM02 and the correlation between RSG and PSORI-CM02 *in vivo* zebrafish models, and the finding suggested that PSORI-CM02, PSORI-CM02 negative drug, and astilbin were able to alleviate CuSO_4_-stimulated inflammatory responses in neutrophil-specific transgenic zebrafish, which could reduce the number of neutrophil recruitment to the injury site. Furthermore, an increase in the number of macrophages engulfing ink was observed, and it demonstrated that they could also promote macrophage phagocytosis. Astilbin had an anti-inflammatory effect, but the concentration of the astilbin monomer was much higher than that of astilbin contained in PSORI-CM02, and even if astilbin (RSG) was removed from PSORI-CM02, it had little effect on anti-inflammatory effect of PSORI-CM02. The above results suggest that other components also have an anti-inflammatory effect in the compound. The specific situation needs further in-depth study.

## Figures and Tables

**Table 1:** “Concentration-mortality” test results after sample treatment (*n* = 30).

Groups	Concentration (*μ*g/mL)	Number of deaths	Mortality (%)	Toxic reactions
Blank control group	—	0/30	0	No abnormal
Model control group	—	0/30	0	No abnormal
A	250	0/30	0	No abnormal
	500	0/30	0	No abnormal
	1000	0/30	0	No abnormal
	1500	0/30	0	No abnormal
	2000	0/30	0	No abnormal
B	250	0/30	0	No abnormal
	500	0/30	0	No abnormal
	1000	0/30	0	No abnormal
	1500	0/30	0	Insensitive to stimulation, more serious than model control group
	2000	0/30	0	Insensitive to stimulation, more serious than model control group
C	125	0/30	0	No abnormal
	250	0/30	0	No abnormal
	500	0/30	0	No abnormal
	1000	0/30	0	No abnormal
	2000	0/30	0	No abnormal

**Table 2:** Effects of experimental groups on zebrafish inflammation (*n* = 30).

Groups	Concentration (*μ*g/mL)	Number of neutrophils (mean +SE)	Inflammation subsidence (%)
Blank control group	—	6 ± 1	—
Model control group	—	28 ± 1	—
Positive control group	80 *μ*M	16 ± 1^*∗∗∗*^	42.9^*∗∗∗*^
A	222	10 ± 1^*∗∗∗*^	64.3^*∗∗∗*^
	667	17 ± 1^*∗∗∗*^	39.3^*∗∗∗*^
	2000	15 ± 1^*∗∗∗*^	46.4^*∗∗∗*^
B	111	10 ± 1^*∗∗∗*^	64.3^*∗∗∗*^
	333	10 ± 1∗∗∗	64.3^*∗∗∗*^
	1000	17 ± 1^*∗∗∗*^	39.3^*∗∗∗*^
C	111	13 ± 1^*∗∗∗*^	53.6^*∗∗∗*^
	333	11 ± 1^*∗∗∗*^	60.7^*∗∗∗*^
	1000	7 ± 1^*∗∗∗*^	75.0^*∗∗∗*^

Compared with model control group: ^*∗∗∗*^*p<*0.001.

**Table 3:** The results of the “concentration-mortality” test after the treatment of the sample (*n* = 30).

Groups	Concentration (*μ*g/mL)	Number of deaths	Mortality (%)
Blank control group	—	0/30	0
Model control group	—	0/30	0
A	250	0/30	0
	500	0/30	0
	1000	7/30	23.3
	1500	14/30	46.7
	2000	29/30	96.7
B	250	0/30	0
	500	0/30	0
	1000	21/30	70.0
	1500	30/30	100
	2000	30/30	100
C	62.5	0/30	0
	125	0/30	0
	250	0/30	0
	500	0/30	0
	1000	0/30	0

**Table 4:** Primary data on the number of macrophages swallowing ink in the zebrafish head of each experimental group (*n* = 30).

Groups	Concentration (*μ*g/mL)	Number of macrophages engulfing ink (mean +SE)	Promotion of macrophage phagocytosis (%)
Blank control group	—	—	—
Model control group	—	18 ± 1	—
Positive control group	15.6	24 ± 1^*∗∗∗*^	33.3^*∗∗∗*^
A	56	22 ± 1^*∗*^	22.2^*∗*^
	167	23 ± 1^*∗∗*^	27.8^*∗∗*^
	500	26 ± 1^*∗∗∗*^	44.4^*∗∗∗*^
B	56	19 ± 1	5.6
	167	24 ± 1^*∗∗*^	33.3^*∗∗*^
	500	23 ± 2^*∗*^	27.8^*∗*^
C	111	20 ± 2	11.1
	333	26 ± 1^*∗∗∗*^	44.4^*∗∗∗*^
	1000	23 ± 1^*∗*^	27.8^*∗*^

Compared with model control group, ^*∗*^*p<*0.05, ^*∗∗*^*p<*0.01, and ^*∗∗∗*^*p<*0.001.

## Data Availability

The data used to support the findings of this study are included within the article.
